# Molecular analysis of a public cross-neutralizing antibody response to SARS-CoV-2

**DOI:** 10.1101/2022.05.17.492220

**Published:** 2022-05-18

**Authors:** Meng Yuan, Yiquan Wang, Huibin Lv, Ian A. Wilson, Nicholas C. Wu

**Affiliations:** 1 Department of Integrative Structural and Computational Biology, The Scripps Research Institute, La Jolla, CA 92037, USA; 2 Department of Biochemistry, University of Illinois at Urbana-Champaign, Urbana, IL 61801, USA; 3 HKU-Pasteur Research Pole, School of Public Health, Li Ka Shing Faculty of Medicine, The University of Hong Kong, Hong Kong SAR, China; 4 The Skaggs Institute for Chemical Biology, The Scripps Research Institute, La Jolla, CA 92037, USA; 5 Center for Biophysics and Quantitative Biology, University of Illinois at Urbana-Champaign, Urbana, IL 61801, USA; 6 Carl R. Woese Institute for Genomic Biology, University of Illinois at Urbana-Champaign, Urbana, IL 61801, USA; 7 Carle Illinois College of Medicine, University of Illinois at Urbana-Champaign, Urbana, IL 61801, USA

## Abstract

As SARS-CoV-2 variants of concerns (VOCs) continue to emerge, cross-neutralizing antibody responses become key towards next-generation design of a more universal COVID-19 vaccine. By analyzing published data from the literature, we report here that the combination of germline genes IGHV2–5/IGLV2–14 represents a public antibody response to the receptor-binding domain (RBD) that potently cross-neutralizes all VOCs to date, including Omicron and its sub-lineages. Detailed molecular analysis shows that the complementarity-determining region H3 sequences of IGHV2–5/IGLV2–14-encoded RBD antibodies have a preferred length of 11 amino acids and a conserved HxIxxI motif. In addition, these antibodies have a strong allelic preference due to an allelic polymorphism at amino-acid residue 54 of IGHV2–5, which locates at the paratope. These findings have important implications for understanding cross-neutralizing antibody responses to SARS-CoV-2 and its heterogenicity at the population level as well as the development of a universal COVID-19 vaccine.

The effectiveness of COVID-19 vaccines has been challenged by the evolution of diverse SARS-CoV-2 variants in the past two years. The recent emergence of Omicron and its sub-lineages BA.2, BA.2.12.1, BA.4, and BA.5 further highlights the urgent need for a more broadly protective vaccine. An ideal COVID-19 vaccine should elicit high titers of neutralizing antibodies that are potent against antigenically distinct variants. However, many potent neutralizing antibodies only have limited cross-reactivity for variants other than the immunizing strain. For example, a major class of antibodies to the receptor-binding domain (RBD) that are encoded by IGHV3–53/3–66 are highly potent against the ancestral Hu-1 strain, but most of them lose their activity against many other variants [1, 2]. Similarly, Beta-specific antibodies can be elicited without cross-neutralizing activity against ancestral or other variants [3]. On the other hand, antibodies to S2 are typically broadly reactive but have weak neutralizing activity [4–6]. Nevertheless, a few RBD antibodies exhibit marked neutralization potency and breadth, as exemplified by those to the RBS-D epitope [2].

One representative RBS-D antibody is LY-CoV1404 (also known as Bebtelovimab), which is a monoclonal therapeutic antibody from Eli Lilly. LY-CoV1404 is encoded by IGHV2–5/IGLV2–14 and can cross-neutralize the ancestral Hu-1 strain as well as all known variants of concern (VOCs), including Omicron and circulating sub-lineages [7, 8]. In fact, the binding mode of LY-CoV1404 is identical to the cross-neutralizing antibody 2–7, which is also encoded by IGHV2–5/IGLV2–14 [9]. More recently, Veesler and colleagues reported another potently cross-neutralizing antibody with similar sequences and binding mode as LY-CoV1404 [10]. As IGHV2–5 was shown to be an important contributor to cross-neutralizing antibody response [11], the observations above stimulated a systematic analysis of IGHV2–5/IGLV2–14-encoded RBD antibodies to SARS-CoV-2.

In our previous study, we assembled a dataset of ~8,000 antibodies to SARS-CoV-2 spike (S) protein [12]. This dataset contains seven IGHV2–5/IGLV2–14-encoded RBD antibodies, including LY-CoV1404, from six different donors [7, 13–17]. In addition, four additional IGHV2–5/IGLV2–14-encoded RBD antibodies were reported in a recent study [18]. Our analysis here is therefore based on a total of 11 IGHV2–5/IGLV2–14-encoded RBD antibodies from at least seven donors. Three of these 11 antibodies have available information for the complete nucleotide sequence, nine have complete amino-acid sequence information, 10 have amino-acid sequence information for the complementarity-determining regions (CDRs) H3 and L3, and four have structure information. Neutralizing data from previous studies have demonstrated that these IGHV2–5/IGLV2–14-encoded RBD antibodies have high cross-neutralizing activity [7, 18–20], some of which remain potent against Omicron (Figure 1A). Previous studies have also shown that they compete with ACE2 for RBD binding [7, 18, 20, 21] (Figure S1).

Next, we performed a structural analysis to uncover the sequence determinants of IGHV2–5/IGLV2–14-encoded antibodies for RBD engagement. For antibody residues, the Kabat numbering scheme is used unless otherwise stated. All four IGHV2–5/IGLV2–14-encoded RBD antibodies with available structural information exhibit the same binding mode to the RBD (Figure 1B). As observed in LY-CoV1404, most amino-acid side chains in the paratope are germline-encoded and form key interactions with the RBD (Figure 1C). For example, germline-encoded V_H_ S32 in the CDR H1 fits into a polar pocket in the RBD. In addition, germline-encoded V_H_ Y52, D54, D56, and R58 in the CDR H2 form an extensive network of H-bonds and electrostatic interactions with the RBD. Furthermore, two key paratope residues in the light chain V_L_ Y32 and Y91 are also germline-encoded. These observations demonstrate that the RBD-binding determinants are encoded in the germline sequences of IGHV2–5 and IGLV2–14. Consistently, several IGHV2–5/IGLV2–14-encoded RBD antibodies have very few somatic hypermutations (SHMs) (Table S1). For example, S24–223 has only one SHM, and COV2–2268 and 2–7 have only four each. Of note, none of their SHMs overlap.

Additional sequence analysis indicated that IGHV2–5/IGLV2–14-encoded RBD antibodies had a strong allelic preference towards IGHV2–5*02. Eight out of 11 IGHV2–5/IGLV2–14-encoded RBD antibodies could be assigned to IGHV2–5*02, while the allele usage for the other three was ambiguous (Figure 1D and Table S1). In contrast, analysis of the B cell repertoire in 13 healthy donors [22, 23] showed that alleles IGHV2–5*01 and IGHV2–5*02 were both commonly used, with a frequency of 33% and 64%, respectively, among all IGHV2–5 antibodies (Figure 1D and Table S2). The lack of IGHV2–5*01 among IGHV2–5/IGLV2–14-encoded RBD antibodies is likely due to an allelic polymorphism at residue 54. IGHV2–5*01 and IGHV2–5*02 have Asn and Asp, respectively, at residue 54. V_H_ D54 in IGHV2–5/IGLV2–14-encoded RBD antibodies plays an important role in RBD binding through a salt bridge with RBD K444 and an H-bond with RBD N450 (Figure 1C). Replacing the Asp at V_H_ residue 54 by Asn would convert the salt bridge with RBD K444 to a H-bond, which would likely significantly weaken the binding energy. Consistently, all eight of the nine IGHV2–5/IGLV2–14-encoded RBD antibodies with sequence information available have an Asp at V_H_ residue 54, whereas the remaining one has a Glu at V_H_ residue 54 (Table S1). These findings provide a mechanistic basis for the allelic preference against IGHV2–5*01, despite its prevalence in the human population. Coincidentally, an almost identical observation was observed in an IGHV2–5-encoded HIV antibody, in which V_H_ D54 results in much stronger binding than V_H_ N54 [24].

Lastly, we analyzed the CDR H3 sequences of the IGHV2–5/IGLV2–14-encoded RBD antibodies. Among 10 IGHV2–5/IGLV2–14-encoded RBD antibodies with CDR H3 sequence information available, eight had a CDR H3 length of 11 amino acids (IMGT numbering) and came from at least five patients (Figure 2A). The CDR H3 sequences from these eight antibodies shared a motif HxIxxI or conserved variations of it, including HxIxxL and HxVxxI (Figures 2A and 2B). The HxIxxI motif consisted of V_H_ H95, I97, and I100 (Kabat numbering) and is uncommon among the CDR H3 sequences of IGHV2–5-encoded antibodies in the human antibody repertoire (Figure 2B). V_H_ H95, I97, and I100 in the HxIxxI motif play critical roles in stabilizing the loop conformation as well as RBD binding (Figure 2C). V_H_ H95 forms two intramolecular H-bonds to stabilize the CDR H3 loop. The first H-bond involves the side chain of V_H_ Y52, which in turn H-bonds with RBD V445 amide nitrogen. The second H-bond involves the backbone carbonyl of V_H_ I100. In addition, V_H_ H95 also forms van der Waals interaction with RBD V445. V_H_ I97 at the tip of the CDR H3 loop inserts into a hydrophobic pocket formed by RBD V445 and P499, as well as the aliphatic portion of RBD N440. V_H_ I100 helps position V_L_ Y91 to interact with RBD V445 and P499. As shown by IgBlast analysis [25], the HxIxxI motif is largely encoded by N-nucleotide addition, although V_H_ I97 may sometimes be encoded by an IGHD gene (Figure 2D). Of note, while CDR H3 of XG005 has 12 amino acids (Figure 2A), it adopts a similar conformation to those with 11 amino acids (Figure S2). Overall, IGHV2–5/IGLV2–14-encoded RBD antibodies with a CDR H3 length of 11 amino acids have convergent CDR H3 sequences, and thus can be classified as a public clonotype.

Due to the continuous evolution of SARS-CoV-2 VOCs, identification of cross-neutralizing human monoclonal antibodies has been a global research focus. IGHV1–58/IGKV3–20-encoded RBD antibodies are perhaps the most well-characterized public antibody clonotype that is cross-neutralizing against multiple SARS-CoV-2 VOCs [3, 12, 26–29]. However, recent studies have shown that many IGHV1–58/IGKV3–20-encoded RBD antibodies have minimal neutralizing activity against Omicron and its sub-lineages due to mutations Q493R and F486V on the RBD [30–32]. In comparison, IGHV2–5/IGLV2–14-encoded RBD antibodies, which mostly retain potency against Omicron and its sub-lineages (Figure 1A) [7, 8, 18–20, 33], have higher neutralization breadth. Since IGHV2–5/IGLV2–14-encoded RBD antibodies are also a public antibody clonotype, they further substantiate the rationale and strategy for development of a more universal COVID-19 vaccine.

Nevertheless, some individuals may have difficulties generating an IGHV2–5/IGLV2–14-encoded RBD antibody response, due to the alleles that they possess (Figure 1D). Since there is no known copy number variation for IGHV2–5 [34], each person should carry two copies of IGHV2–5 in the genome. If both copies are IGHV2–5*01 allele, the person may not have the suitable B cell germline clone to produce a IGHV2–5/IGLV2–14-encoded RBD antibody response. In fact, donor 112 in the 13 healthy donors that were analyzed in this study is very likely to be IGHV2–5*01 homozygous, since 94% of its IGHV2–5-encoded antibodies were assigned to IGHV2–5*01 (Table S2). Moreover, the conserved HxIxxI motif in CDR H3 of IGHV2–5/IGLV2–14-encoded RBD antibodies is mostly encoded by random N-nucleotide addition. As a result, B cell germline clones that can produce IGHV2–5/IGLV2–14-encoded RBD antibodies may be relatively rare. These results may provide a genetic basis for heterogenicity in the cross-neutralizing antibody response among different individuals. While allelic preference has previously been described for neutralizing antibodies to other viruses [24, 35–37], its clinical implications for COVID-19 remain to be fully explored.

## MATERIALS AND METHODS

### Dataset collection

The information on antibodies S24–223, P2B-1E4, 2–7, LY-CoV1404, XG005, XG031, and COV2–2268 were compiled in our previous study [12], whereas the information on XGv042, XGv264, XGv265, and XGv266 were compiled in CoV-AbDab [38]. Neutralization data of each monoclonal antibody were collected from the original papers (Table S1). Somatic hypermutations were identified by IgBlast [25].

### Allele assignment of IGHV2–5/IGLV2–14-encoded RBD antibodies

For antibodies P2B-1E4, XG005, and XG031, the allele information was obtained from the original publications [14, 16]. For other antibodies, IgBlast was used to assign the allele of each antibody [25]. Nucleotide sequence, if available, was used as input for IgBlast. Otherwise, protein sequence was used. If an antibody showed equally likely to be encoded by two or more alleles, the allele assignment would be classified as “ambiguous”. All “ambiguous” allele assignments in this study came from antibodies that do not have nucleotide sequence information available, namely XGv264, XGv265, and XGv266. Of note, while IgBlast showed that XGv266 was equally likely to be encoded by IGHV2–5*01 and IGHV2–5*02, we postulated that XGv266 should be assigned to IGHV2–5*02 at the nucleotide level. Specifically, XGv266 had a Glu at V_H_ residue 54, which was one nucleotide change from the Asp codon used (IGHV2–5*02) but two nucleotides away from Asn (IGHV2–5*01). However, IgBlast did not utilize codon information for allele assignment when the amino-acid sequence was used as input.

### Analysis of allele usage in published antibody repertoire

Published antibody repertoire sequencing datasets from 13 healthy donors [22, 23] were downloaded from cAb-Rep [39]. Putative germline gene alleles for each antibody sequence in these repertoire sequencing datasets from healthy donors were identified by IgBLAST [25].

### Analysis of CDR H3 sequences

Sequence alignment was performed using MAFFT [40]. Antibody sequences in the human antibody repertoire were downloaded from the Observed Antibody Space [41]. IGHV2–5 antibodies as well as their CDR H3 sequences were identified using IgBLAST [25]. Sequence logos were generated by WebLogo [42]. Putative germline sequences and segments in the V-D-J junctions were identified by IgBLAST [25].

### Code Availability

Custom codes for all analyses have been deposited to https://github.com/nicwulab/IGHV2-5_RBD_Abs.

## Supplementary Material

Supplement 1

Supplement 2

1

## Figures and Tables

**Figure 1.**
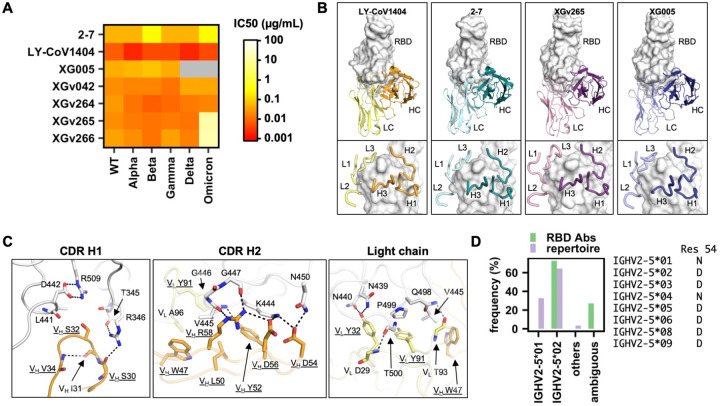
IGHV2–5/IGLV2–14 is a public antibody response with strong allelic preference. **(A)** The half maximal inhibitory concentration (IC_50_) of IGHV2–5/IGLV2–14-encoded RBD antibodies against different SARS-CoV-2 VOCs in pseudovirus assays. Data were taken from previous studies [7, 18–20]. **(B)** Four IGHV2–5/IGLV2–14-encoded RBD antibodies have structure information available. Their binding modes to RBD (white surface) are compared. Upper panels: heavy chain (HC) and light chain (LC) of each antibody are shown. Bottom panels: zoom-in views with six CDR loops of each antibody shown. LY-CoV1404: PDB 7MMO [7]. 2–7: PDB 7LSS [21]. XGv265: PDB 7WEE [18]. XG005: PDB 7V26 [20]. **(C)** Key interactions between LY-CoV1404 and RBD are shown. Hydrogen bonds and salt bridges are represented by black dashed lines. All germline-encoded residues are underlined. Heavy chain is in orange, light chain in yellow, and RBD is in white. **(D)** IGHV allele usage of the 11 IGHV2–5/IGLV2–14-encoded RBD antibodies (RBD Abs) is compared with that of IGHV2–5-encoded antibodies in published repertoire sequencing datasets from 13 healthy donors [22, 23]. The amino-acid identity at residue 54 of each IGHV2–5 allele is indicated.

**Figure 2.**
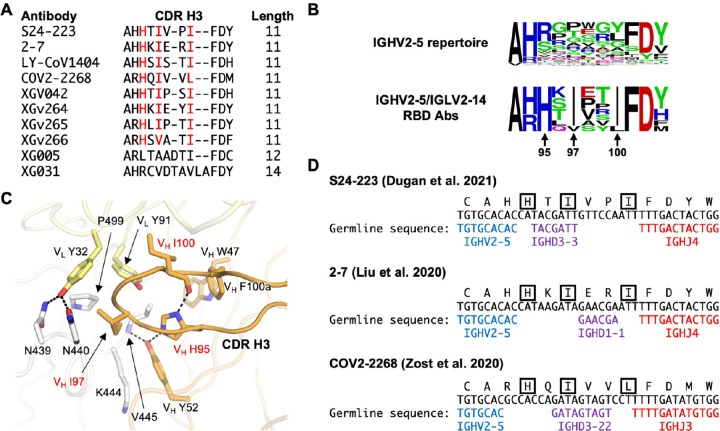
HxIxxI is a common motif in IGHV2–5/IGLV2–14-encoded RBD antibodies. **(A)** CDR H3 sequences (IMGT numbering) from IGHV2–5/IGLV2–14-encoded RBD antibodies are aligned. Residues of interest are highlighted in red. **(B)** CDR H3 sequences (IMGT numbering) of IGHV2–5/IGLV2–14-encoded RBD antibodies and IGHV2–5-encoded antibodies in the human antibody repertoire are shown as sequence logos. Only those antibodies with a CDR H3 length of 11 amino acids are included in this analysis. Residues of interest are labeled. Sequences of IGHV2–5-encoded antibodies in the human antibody repertoire were downloaded from the Observed Antibody Space [41]. A total of 9,197 IGHV2–5-encoded antibodies in the human antibody repertoire were analyzed here. Of note, while Kabat numbering was used for the residue position, IMGT numbering was used for defining CDR H3 length. **(C)** Interaction between the CDR H3 of LY-CoV1404 and RBD is shown. PDB 7MMO [7]. Hydrogen bonds are represented by black dashed lines. Heavy chain is in orange, light chain in yellow, and RBD is in white. Residues of interest are highlighted in red. **(D)** Amino-acid and nucleotide sequences of the V-D-J junction of three IGHV2–5/IGLV2–14-encoded RBD antibodies are shown. Putative germline sequences and segments are indicated. Residues of interest are boxed.

## References

[1] Zhang Q, Potent and protective IGHV3–53/3–66 public antibodies and their shared escape mutant on the spike of SARS-CoV-2. Nat Commun 12, 4210 (2021) 34244522 10.1038/s41467-021-24514-w PMC8270942

[2] Yuan M, Structural and functional ramifications of antigenic drift in recent SARS-CoV-2 variants. Science 373, 818–823 (2021) 34016740 10.1126/science.abh1139 PMC8284396

[3] Reincke SM, SARS-CoV-2 Beta variant infection elicits potent lineage-specific and cross-reactive antibodies. Science 375, 782–787 (2022) 35076281 10.1126/science.abm5835 PMC8939768

[4] Zhou P, A human antibody reveals a conserved site on beta-coronavirus spike proteins and confers protection against SARS-CoV-2 infection. Sci Transl Med 14, eabi9215 (2022) 35133175 10.1126/scitranslmed.abi9215 PMC8939767

[5] Pinto D, Broad betacoronavirus neutralization by a stem helix-specific human antibody. Science 373, 1109–1116 (2021) 34344823 10.1126/science.abj3321 PMC9268357

[6] Li W, Structural basis and mode of action for two broadly neutralizing antibodies against SARS-CoV-2 emerging variants of concern. Cell Rep 38, 110210 (2022) 34971573 10.1016/j.celrep.2021.110210 PMC8673750

[7] Westendorf K, LY-CoV1404 (bebtelovimab) potently neutralizes SARS-CoV-2 variants. Cell Reports 39, 110812 (2022) 35568025 10.1016/j.celrep.2022.110812 PMC9035363

[8] Cao Y, BA.2.12.1, BA.4 and BA.5 escape antibodies elicited by Omicron infection. bioRxiv, doi: 10.1101/2022.04.30.489997, (2022) PMC9385493 35714668

[9] Kramer KJ, Potent neutralization of SARS-CoV-2 variants of concern by an antibody with an uncommon genetic signature and structural mode of spike recognition. Cell Rep 37, 109784 (2021) 34592170 10.1016/j.celrep.2021.109784 PMC8443366

[10] Park YJ, Imprinted antibody responses against SARS-CoV-2 Omicron sublineages. bioRxiv, doi: 10.1101/2022.05.08.491108, (2022) PMC12945441 36264829

[11] Andreano E, Hybrid immunity improves B cells and antibodies against SARS-CoV-2 variants. Nature 600, 530–535 (2021) 34670266 10.1038/s41586-021-04117-7 PMC8674140

[12] Wang Y, A large-scale systematic survey reveals recurring molecular features of public antibody responses to SARS-CoV-2. Immunity, doi: 10.1016/j.immuni.2022.03.019, (2022) PMC8947961 35397794

[13] Dugan HL, Profiling B cell immunodominance after SARS-CoV-2 infection reveals antibody evolution to non-neutralizing viral targets. Immunity 54, 1290–1303.e1297 (2021) 34022127 10.1016/j.immuni.2021.05.001 PMC8101792

[14] Ju B, Human neutralizing antibodies elicited by SARS-CoV-2 infection. Nature 584, 115–119 (2020) 32454513 10.1038/s41586-020-2380-z

[15] Liu L, Potent neutralizing antibodies directed to multiple epitopes on SARS-CoV-2 spike. Nature 584, 450–456 (2020) 32698192 10.1038/s41586-020-2571-7

[16] Zhou Y, Enhancement versus neutralization by SARS-CoV-2 antibodies from a convalescent donor associates with distinct epitopes on the RBD. Cell Rep 34, 108699 (2021) 33485405 10.1016/j.celrep.2021.108699 PMC7802522

[17] Zost SJ, Rapid isolation and profiling of a diverse panel of human monoclonal antibodies targeting the SARS-CoV-2 spike protein. Nat Med 26, 1422–1427 (2020) 32651581 10.1038/s41591-020-0998-x PMC8194108

[18] Wang K, Memory B cell repertoire from triple vaccinees against diverse SARS-CoV-2 variants. Nature 603, 919–925 (2022) 35090164 10.1038/s41586-022-04466-x PMC8967717

[19] Liu L, Striking antibody evasion manifested by the Omicron variant of SARS-CoV-2. Nature 602, 676–681 (2022) 35016198 10.1038/s41586-021-04388-0

[20] Liu Z, An ultrapotent pan-β-coronavirus lineage B (β-CoV-B) neutralizing antibody locks the receptor-binding domain in closed conformation by targeting its conserved epitope. Protein Cell, doi: 10.1007/s13238-021-00871-6, (2021) PMC8458794 34554412

[21] Cerutti G, Structural basis for accommodation of emerging B.1.351 and B.1.1.7 variants by two potent SARS-CoV-2 neutralizing antibodies. Structure 29, 655–663.e654 (2021) 34111408 10.1016/j.str.2021.05.014 PMC8188728

[22] Briney B, Inderbitzin A, Joyce C, Burton DR. Commonality despite exceptional diversity in the baseline human antibody repertoire. Nature 566, 393–397 (2019) 30664748 10.1038/s41586-019-0879-y PMC6411386

[23] Soto C, High frequency of shared clonotypes in human B cell receptor repertoires. Nature 566, 398–402 (2019) 30760926 10.1038/s41586-019-0934-8 PMC6949180

[24] Alam SM, Differential reactivity of germ line allelic variants of a broadly neutralizing HIV-1 antibody to a gp41 fusion intermediate conformation. J Virol 85, 11725–11731 (2011) 21917975 10.1128/JVI.05680-11 PMC3209283

[25] Ye J, Ma N, Madden TL, Ostell JM. IgBLAST: an immunoglobulin variable domain sequence analysis tool. Nucleic Acids Res 41, W34–40 (2013) 23671333 10.1093/nar/gkt382 PMC3692102

[26] Dejnirattisai W, The antigenic anatomy of SARS-CoV-2 receptor binding domain. Cell 184, 2183–2200.e2122 (2021) 33756110 10.1016/j.cell.2021.02.032 PMC7891125

[27] Robbiani DF, Convergent antibody responses to SARS-CoV-2 in convalescent individuals. Nature 584, 437–442 (2020) 32555388 10.1038/s41586-020-2456-9 PMC7442695

[28] Tortorici MA, Ultrapotent human antibodies protect against SARS-CoV-2 challenge via multiple mechanisms. Science 370, 950–957 (2020) 32972994 10.1126/science.abe3354 PMC7857395

[29] Wang L, Ultrapotent antibodies against diverse and highly transmissible SARS-CoV-2 variants. Science 373, eabh1766 (2021) 34210892 10.1126/science.abh1766 PMC9269068

[30] Dong J, Genetic and structural basis for SARS-CoV-2 variant neutralization by a two-antibody cocktail. Nat Microbiol 6, 1233–1244 (2021) 34548634 10.1038/s41564-021-00972-2 PMC8543371

[31] Starr TN, SARS-CoV-2 RBD antibodies that maximize breadth and resistance to escape. Nature 597, 97–102 (2021) 34261126 10.1038/s41586-021-03807-6 PMC9282883

[32] FDA. Fact sheet for healthcare providers: emergency ese authorization for Evusheld (tixagevimab co-packaged with cilgavimab). doi, (2021)

[33] Yamasoba D, Sensitivity of novel SARS-CoV-2 Omicron subvariants, BA.2.11, BA.2.12.1, BA.4 and BA.5 to therapeutic monoclonal antibodies. bioRxiv, doi: 10.1101/2022.05.03.490409, (2022) PMC9179126 35690075

[34] Lefranc MP, Lefranc G. Immunoglobulins or antibodies: IMGT bridging genes, structures and functions. Biomedicines 8, 319 (2020) 10.3390/biomedicines8090319 PMC7555362 32878258

[35] Ying T, Junctional and allele-specific residues are critical for MERS-CoV neutralization by an exceptionally potent germline-like antibody. Nat Commun 6, 8223 (2015) 26370782 10.1038/ncomms9223 PMC4571279

[36] Lang S, Antibody 27F3 broadly targets influenza A group 1 and 2 hemagglutinins through a further variation in V_H_1–69 antibody orientation on the HA stem. Cell Rep 20, 2935–2943 (2017) 28930686 10.1016/j.celrep.2017.08.084 PMC5679313

[37] Avnir Y, IGHV1–69 polymorphism modulates anti-influenza antibody repertoires, correlates with IGHV utilization shifts and varies by ethnicity. Sci Rep 6, 20842 (2016) 26880249 10.1038/srep20842 PMC4754645

[38] Raybould MIJ, Kovaltsuk A, Marks C, Deane CM. CoV-AbDab: the coronavirus antibody database. Bioinformatics 37, 734–735 (2021) 32805021 10.1093/bioinformatics/btaa739 PMC7558925

[39] Guo Y, cAb-Rep: a database of curated antibody repertoires for exploring antibody diversity and predicting antibody prevalence. Front Immunol 10, 2365 (2019) 31649674 10.3389/fimmu.2019.02365 PMC6794461

[40] Katoh K, Standley DM. MAFFT multiple sequence alignment software version 7: improvements in performance and usability. Mol Biol Evol 30, 772–780 (2013) 23329690 10.1093/molbev/mst010 PMC3603318

[41] Kovaltsuk A, Observed Antibody Space: a resource for data mining next-generation sequencing of antibody repertoires. J Immunol 201, 2502–2509 (2018) 30217829 10.4049/jimmunol.1800708

[42] Crooks GE, Hon G, Chandonia JM, Brenner SE. WebLogo: a sequence logo generator. Genome Res 14, 1188–1190 (2004) 15173120 10.1101/gr.849004 PMC419797

[43] Lan J, Structure of the SARS-CoV-2 spike receptor-binding domain bound to the ACE2 receptor. Nature 581, 215–220 (2020) 32225176 10.1038/s41586-020-2180-5

