# Supplementary material for: Molecular analysis of a public cross-neutralizing antibody response to SARS-CoV-2
Source: bioRxiv. 2022 May 18:2022.05.17.492220. Preprint. [Version 1] doi: 10.1101/2022.05.17.492220 (PMC9128778; doi:10.1101/2022.05.17.492220)
Supplement: 1 [file NIHPP2022.05.17.492220V1-supplement-1.pdf]

bioRxiv preprint doi: https://doi.org/10.1101/2022.05.17.492220; this version posted May 18, 2022. The copyright holder for this preprint (which was not certified by peer review) is the author/funder, who has granted bioRxiv a license to display the preprint in perpetuity. It is made available under aCC-BY 4.0 International license.

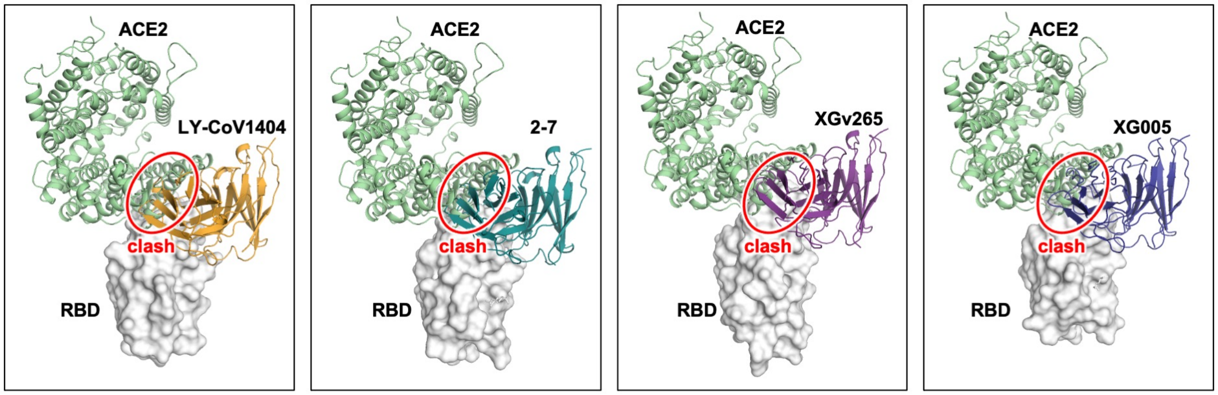


**Figure S1. IGHV2-5/IGLV2-14 antibodies would clash with ACE2 binding.** Structures of antibody/RBD complexes are superimposed onto the RBD/ACE2 complex structure (PDB 6M0J) [43]. RBD is represented by a white surface. ACE2 is shown as green cartoon. LY-CoV1404: PDB 7MMO [7]. 2-7: PDB 7LSS [21]. XGv265: PDB 7WEE [18]. XG005: PDB 7V26 [20].

bioRxiv preprint doi: https://doi.org/10.1101/2022.05.17.492220; this version posted May 18, 2022. The copyright holder for this preprint (which was not certified by peer review) is the author/funder, who has granted bioRxiv a license to display the preprint in perpetuity. It is made available under aCC-BY 4.0 International license.

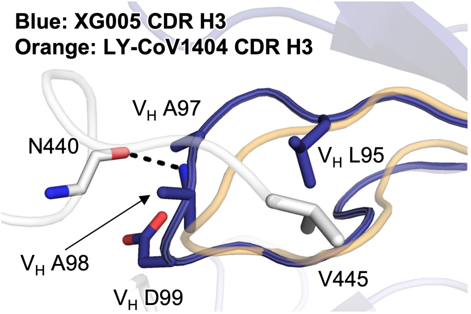


**Figure S2. Interactions between RBD and CDR H3 of XG005.** A cryo-EM structure of SARS-CoV-2 spike protein in complex with XG005 (PDB 7V26) that was reported in a previous study [20] is shown. A hydrogen bond between XG005 and the RBD is represented by a black dashed line. The CDR H3 of LY-CoV1404 (PDB 7MMO) [7] is also shown here as a transparent orange cartoon to demonstrate the relative positions of the CDR H3 loops from these two antibodies after superimposition.
